# People living with HIV with the Omicron variant infection have milder COVID-19 symptoms: results from a cross-sectional study

**DOI:** 10.1186/s12981-024-00633-4

**Published:** 2024-08-10

**Authors:** Yuting Tan, Songjie Wu, Fangzhao Ming, Jie Liu, Gifty Marley, Aiping Yu, Yanhe Luo, Shi Zou, Wei Guo, Weiming Tang, Ke Liang

**Affiliations:** 1 Department of Infectious Diseases, Zhongnan Hospital of Wuhan University, Wuhan, China; 2 Wuhan Research Center for Infectious Diseases and Cancer, Chinese Academy of Medical Sciences, Wuhan, China; 3 Department of Nosocomial Infection Management, Zhongnan Hospital of Wuhan University, Wuhan, China; 4 Wuchang District Center for Disease Control and Prevention, Wuhan, China; 5 The University of North Carolina at Chapel Hill Project-China, Guangzhou, China; 6 Dongxihu District Center for Disease Control and Prevention, Wuhan, China; 7 Wuhan Jinyintan Hospital, Wuhan, China; 8 Department of Pathology, Zhongnan Hospital of Wuhan University, Wuhan, China; 9 Department of Pathology, School of Basic Medical Sciences, Wuhan University, Wuhan, China; 10 Guangdong No. 2 Provincial People’s Hospital, Guangzhou, China; 11 Hubei Engineering Center for Infectious Disease Prevention, Control and Treatment, 169 Donghu Road, Wuchang District, Wuhan, Hubei Province China

**Keywords:** HIV, COVID-19, Omicron, Symptom prevalence, Risk factor

## Abstract

**Background:**

China braces for coronavirus disease 2019 (COVID-19) surge after adjusting the “zero COVID” strategy. We aimed to evaluate and compare the prevalence of clinical symptoms of the Omicron variant infection among people living with HIV (PLWH) and HIV-free people.

**Methods:**

A cross-sectional study was conducted in Wuchang District, Wuhan, Hubei Province, in December 2022 by a self-administered online survey during the Omicron wave. Participants aged ≥ 18 years with confirmed severe acute respiratory syndrome coronavirus 2 (SARS-CoV-2) diagnosis were recruited. PLWH managed by the local healthcare system were recruited, while HIV-free people were recruited by sending out online surveys through WeChat. We compared the prevalence of clinical symptoms of COVID-19 between PLWH and HIV-free people, and factors associated with symptom occurrence among PLWH were accessed.

**Results:**

Total, 687 PLWH and 1222 HIV-free people were enrolled. After adjusting sex, age, body mass index, comorbidities and COVID-19 vaccination status, the prevalences of all symptoms, including higher degree and long duration of fever (aOR 0.51, 95%CI 0·42 − 0·61; aOR 0.52, 95%CI 0·43 − 0·63), were significantly lower among PLWH than among HIV-free people. Among PLWH, CD4^+^ T lymphocyte count (CD4 count) between 350 ~ 499 cells/µL and detectable HIV viral load (HIV-VL) were associated with significantly decreased risks of fever (aOR 0·63, 95%CI 0·40 − 0·97; aOR 0·56, 95%CI 0·33 − 0·94), headache (aOR 0·61, 95%CI 0·41 − 0·91; aOR 0·55, 95%CI 0·34 − 0·92) and muscle soreness (aOR 0·57, 95%CI 0·39 − 0·84; aOR 0·57, 95%CI 0·39 − 0·84). No apparent association between the symptoms prevalence and three/four doses of inactivated COVID-19 vaccination among PLWH was observed; both males and older age were associated with significantly decreased risks of nasal congestion/runny nose (aOR 0·52, 95%CI 0·32 − 0·82; aOR 0·97, 95%CI 0·96 − 0·99) and headache (aOR 0·58, 95%CI 0·36 − 0·92; aOR 0·96, 95%CI 0·95 − 0·98); older age was associated with significantly decreased risks of higher degree of fever (aOR 0·97, 95%CI 0·95 − 0·98).

**Conclusions:**

PLWH have significantly milder symptoms of the Omicron variant infection than HIV-free people. PLWH who are male, older, have low CD4 count, and detectable HIV-VL have reduced occurrence of COVID-19 symptoms. However, continuous monitoring should be conducted among PLWH during the COVID-19 pandemic.

**Supplementary Information:**

The online version contains supplementary material available at 10.1186/s12981-024-00633-4.

## Background

Increased coronavirus disease 2019 (COVID-19) mortality caused by severe acute respiratory syndrome coronavirus 2 (SARS-CoV-2) infection has been reported among people living with HIV (PLWH). Our previous study showed that PLWH with SARS-CoV-2 infection had an increased mortality risk compared with HIV-free people [1]. However, limited data also suggested that PLWH had a comparable prevalence of symptomatic SARS-CoV-2 infection, disease course, and COVID-19 disease severity compared to HIV-free people during previous SARS-CoV-2 waves [2–4].

Omicron, a SARS-CoV-2 variant with high transmission and immune evasion ability, has posed a challenge to global epidemic control and has increasingly been dominant in China since 2022. Compared with previous variants of concern, weakened pathogenicity of the Omicron variant has been reported [5]. In Africa, decreased odds of hospitalization (2·4%~4·9%) and severe disease from the Omicron variant infection were observed compared with non-Omicron variants infection and the Delta variants infection [6, 7]. A previous small sample size study in China found that persons infected with the Omicron variant had milder clinical symptoms and fewer complications than persons infected with the wild type and Delta variants [8]. After removing the “zero COVID” strategy on December 7, 2022, there was a surge in the COVID-19 pandemic with the Omicron variant infection in China. It is estimated that the cumulative infection attack rate in Beijing reached 76% on December 22, 2022, which indicated that more than half of the Chinese people were infected with the Omicron variant in less than a month [9].

Although relevant studies abroad sugges*t*ed that most patients infected with the Omicron variant were not hospitalized [6, 7], it is still unclear how SARS-CoV-2 impacted PLWH during the Omicron wave in China. We have previously investigated the clinical characteristics of HIV and SARS-CoV-2 co-infection during the early SARS-CoV-2 wave in Wuhan, Hubei Province, in 2020 and accessed the efficacy and safety of two and three doses of inactivated COVID-19 vaccines among PLWH [10–13]. This cross-sectional study aims to extend our previous work by investigating and comparing the prevalence of clinical symptoms of the Omicron variant infection among PLWH and HIV-free people.

## Methods

### Study design and participants

An online cross-sectional survey was conducted in Wuchang District, Wuhan, Hubei Province, in December 2022. PLWH and HIV-free people aged ≥ 18 years old, at the time or within two weeks of acute SARS-CoV-2 infection and volunteered to participate in this survey were eligible. All 1,064 adults with confirmed HIV infection in Wuchang District managed by the Wuchang district center for disease control and prevention (CDC) were invited to the self-administered online survey via WeChat. HIV-free adults were recruited from the same study area by sending out online surveys through WeChat. Participants who had no complete response to the online survey were excluded.

SARS-CoV-2 infection was divided into symptomatic infection and asymptomatic infection. Symptomatic infection was defined as a patient having related symptoms of SARS-CoV-2 infection and positive SARS-CoV-2 nucleic acid testing or rapid antigen test. Asymptomatic infection was defined as having no related symptoms of SARS-CoV-2 infection and a positive SARS-CoV-2 nucleic acid testing or rapid antigen test. SARS-CoV-2 infection-related symptoms included fever, nasal congestion/runny nose, sore/dry throat, headache, cough, chest pain, chest tightness, fatigue, muscle soreness, decreased/loss of smell, decreased/lost taste, diarrhea, and conjunctivitis.

### Questionnaire and data collection

Two separate self-administered online questionnaires hosted on the WeChat-online survey platform named Wenjuanxing (www.wjx.cn) were designed for PLWH and HIV-free people with SARS-CoV-2 infection, respectively. Self-administered online questionnaires were distributed to all participants one by one. Both self-administered online surveys had the same questions, including sex, age, height, weight, comorbidities (having comorbidities, none), HIV status, COVID-19 vaccination status (unvaccinated, one/two doses, three/four doses), diagnostic method of SARS-CoV-2 infection (positive SARS-CoV-2 nucleic acid testing, positive SARS-CoV-2 rapid antigen test), symptoms of SARS-CoV-2 infection including degree of fever (< 37·3℃, 37·3–38℃, 38·1–39℃, ≥ 39·1℃) and duration of fever (0 days, ≤ 3 days, 3–5 days, > 5 days). The confirmation of HIV status in the HIV-free group were depended on the participants’ self-report. Questions on recent CD4^+^ T lymphocyte count (CD4 count, 0-199 cells/µL, 200–349 cells/µL, 350–499 cells/µL, ≥ 500 cells/µL, unknown), HIV viral load (HIV-VL, undetectable, detectable, unknown) and antiretroviral therapy (ART) status (on ART, none) were included in the self -administered online questionnaire for PLWH.

### Outcomes

The primary outcome was the adjusted odds ratios (aORs) of COVID-19 symptoms occurrence at the time or within two weeks of acute SARS-CoV-2 infection among PLWH after adjusting the confounding factors, including age, sex, body mass index (BMI), comorbidities, and COVID-19 vaccination status, and HIV-free people with COVID-19 were as references. The secondary outcome was the aORs of sex, age, BMI, comorbidities, ART status, CD4 T count, HIV viral load, and COVID-19 vaccination status associated with all COVID-19 symptoms, including the higher degree and longer duration of fever, among PLWH.

### Statistical analysis

SPSS version 26·0 (IBM Corp, Armonk, New York, United States) was used for data analysis. Continuous variables were presented as mean and standard deviation (normally distributed) or median with the 25th to 75th interquartile range (IQR) (skewed distribution). Categorical variables were denoted as counts and proportions (%). Continuous variables conformed to the normal distribution were analyzed by group t-test and non-parametric rank sum test. The chi-square test or Kruskal-Wallis rank sum test was used to compare the group counts data. Multivariable logistic regression analysis was conducted to compare the prevalence of symptoms (except degree and duration of fever) between PLWH and HIV-free people with SARS-CoV-2 infection, while the confounding factors, including age, sex, BMI, comorbidities, and COVID-19 vaccination status, were adjusted. The factors associated with the occurrence of the symptoms (except degree and duration of fever) among PLWH with SARS-CoV-2 infection were also conducted by multivariable logistic regression analysis. The generalized linear regression model was performed to compare the prevalence of the higher degree and longer duration of fever between PLWH and HIV-free people with SARS-CoV-2 infection, and examine factors associated with the higher degree and longer duration of fever among PLWH with SARS-CoV-2 infection. Statistical significance was defined as a two-sided p-value < 0.05.

## Results

### Participants’ characteristics

Of all 1,064 adult PLWH in Wuchang District, 720 with confirmed SARS-CoV-2 infection voluntarily participated in the self-administered online questionnaire survey. After 33 cases without a complete response to the online questionnaire were excluded, finally 687 eligible PLWH were enrolled. A total of 1,310 HIV-free adults from Wuchang District voluntarily participated in the self-administered online questionnaire survey. After excluding 88 cases who had no complete response to the online questionnaire, 1,222 eligible HIV-free people were enrolled. The distributions of sex, age, and BMI were significantly different between the two groups. Compared with HIV-free people, the proportion of having comorbidities was significantly higher, and the proportion of three/four doses of COVID-19 vaccination was lower among PLWH (Table 1).

**Table 1:** Characteristics of PLWH and HIV-free people with SARS-CoV-2 infection in Wuhan, Hubei Province, China (*N* = 1,909)

	PLWH (*n* = 687)	HIV-free (*n* = 1222)	*p* value	
Male	580 (84.4%)	484 (39.6%)	< 0.001	
Age, years, median (IQR)	34 (29–42)	35 (30–44)	0.006	
BMI, kg/m^2^, median (IQR)	20.05 (20.24–23.87)	22.40 (20.57–24.77)	< 0.001	
COVID-19 vaccination			< 0.001	
Unvaccinated	68 (9.9%)	42 (3.4%)		
One dose/two doses	150 (21.8%)	234 (19.1%)		
Three doses/four doses^§^	469 (68.3%)	946 (77.4%)		
Comorbidities	217 (31.6%)	152 (12.4%)	< 0.001	
Chronic pulmonary diseases	33 (4.8%)	18 (1.5%)		
Diabetes	17 (2.4%)	17 (1.4%)		
Chronic kidney diseases	12 (1.7%)	4 (0.3%)		
Chronic liver diseases	20 (2.9%)	29 (2.4%)		
Cardiovascular and cerebrovascular diseases	39 (5.6%)	73 (6.0%)		
Autoimmune disease	128 (18.6%)	21 (1.7%)		
Cancer	5 (0.7%)	13 (1.1%)		
CD4 T count, cells/µL				
0-199	47 (6.8%)	··	··	
200–349	91 (13.2%)	··	··	
350–499	179 (26.1%)	··	··	
≥ 500	269 (39.2%)	··	··	
Unknown	101 (14.7%)	··	··	
HIV viral load				
Undetectable	483 (70.3%)	··	··	
Detectable	81 (11.8%)	··	··	
Unknown	123 (17.9%)	··	··	
On ART	667 (97.1%)	··	··	
Symptoms of SARS-CoV-2 infection				
Any symptom	604 (87.9%)	1162 (95.1%)	< 0.001	
Fever	501 (72.9%)	1024 (83.8%)	< 0.001	
Nasal congestion/runny nose	401 (58.4%)	877 (71.8%)	< 0.001	
Sore/dry throat	422 (61.4%)	896 (73.3%)	< 0.001	
Headache	408 (59.4%)	826 (67.6%)	< 0.001	
Cough	423 (61.6%)	922 (75.5%)	< 0.001	
Chest pain	68 (9.9%)	165 (13.5%)	0.021	
Chest tightness	76 (11.1%)	214 (17.5%)	< 0.001	
Fatigue	307 (44.7%)	740 (60.6%)	< 0.001	
Muscle soreness	376 (54.7%)	832 (68.1%)	< 0.001	
Decreased/lost smell	153 (22.3%)	439 (35.9%)	< 0.001	
Decreased or lost taste	203 (29.5%)	520 (42.6%)	< 0.001	
Diarrhea	123 (17.9%)	296 (24.2%)	0.001	
Conjunctivitis	12 (1.7%)	38 (3.1%)	0.074	
Degree of fever			< 0.001	
< 37.3℃	186 (27.1%)	199 (16.3%)	··	
37.3–38.0℃	122 (17.8%)	188 (15.4%)	··	
38.1–39.0℃	276 (40.2%)	542 (44.4%)	··	
≥ 39.1℃	103 (15.0%)	293 (24.0%)	··	
Duration of fever			< 0.001	
No fever	186 (27.1%)	199 (16.3%)	··	
≤ 3 days	403 (58.7%)	746 (61.0%)	··	
3–5 days	84 (12.2%)	255 (20.9%)	··	
>5 days	14 (2.0%)	22 (1.8%)	··	

^§^ Four doses COVID-19 vaccines refers to three doses of inactivated COVID-19 vaccines plus one dose of recombinant adenovirus type 5 (AD5)-vectored COVID-19 vaccine. Data are median (IQR) or n (%). PLWH = people living with HIV. BMI = body mass index. ART = antiretroviral therapy

The clinical presentations of the Omicron variant infection among PLWH and HIV-free people were summarized (Table 1). Totally, 87.9% of PLWH reported at least one symptom, while this was 95.1% among HIV-free participants. Fever (72·9%), cough (61·6%), and pharyngula/dry pharynx (61·4%) were the most common symptoms reported among PLWH. Two-fifth (40·2%) of PLWH had a moderate fever (38·1 ~ 39℃), and more than half of PLWH had a fever duration of fewer than three days.

### Comparison of symptoms prevalence between PLWH and HIV-free people

**Fig. 1:**
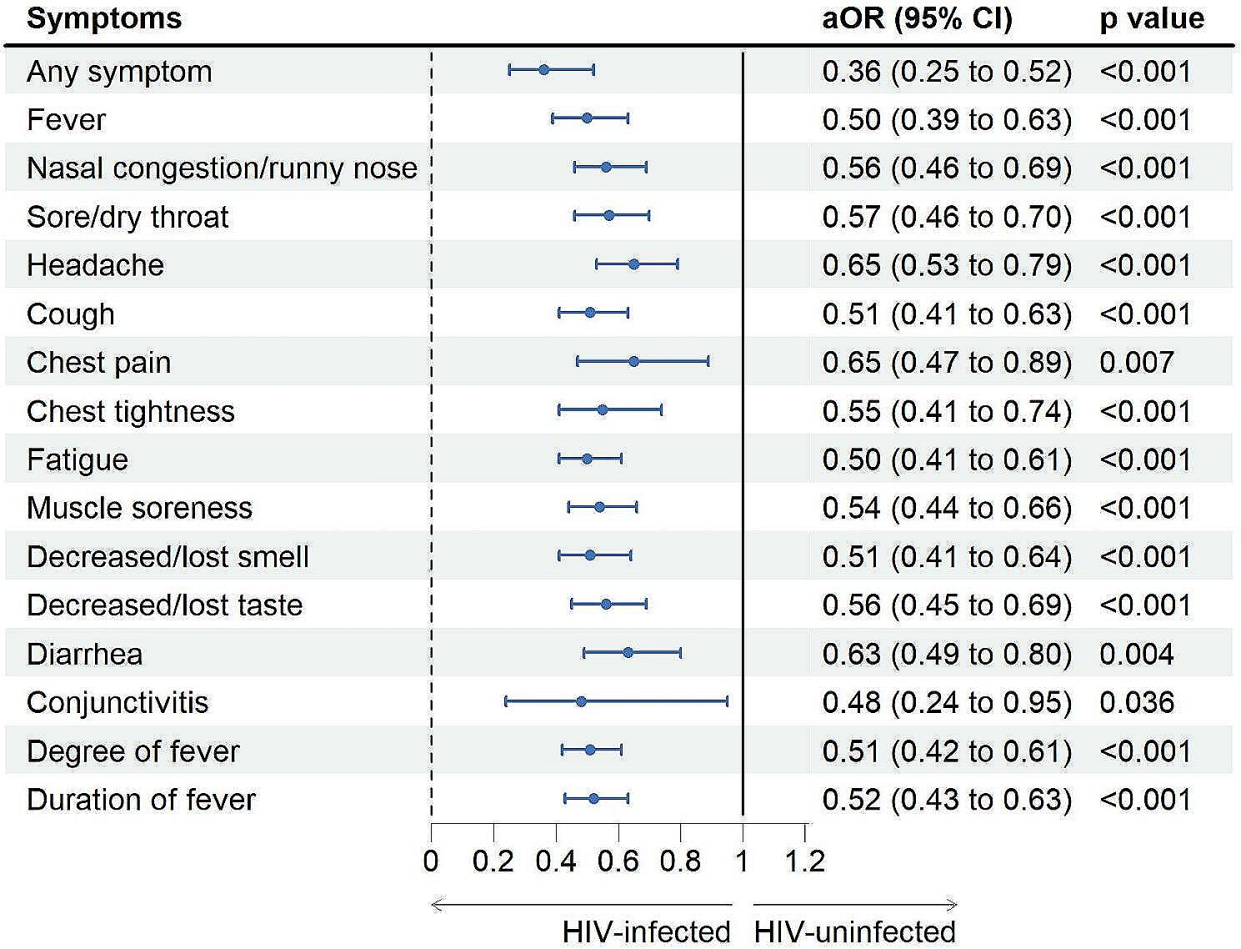
Risks of clinical symptoms occurrence among people living with HIV (PLWH) with SARS-CoV-2 infection (HIV-free people with SARS-CoV-2 infection were as references)

After adjusting the confounding factors, including age, sex, BMI, comorbidities and COVID-19 vaccination status, the prevalence of any symptom was significantly lower among PLWH than among HIV-free people with the Omicron variant infection (aOR 0.36, 95%CI 0.25–0.52). The prevalence of fever (aOR 0.50, 95%CI 0.39–0.63) was significantly lower among PLWH than among HIV-free people. All other symptoms, including nasal congestion/runny nose (aOR 0·56, 95%CI 0·46 − 0·69), sore/dry throat (aOR 0·57, 95%CI 0·46 − 0·70), headache (aOR 0·65, 95%CI 0·53 − 0·79), cough (aOR 0·51, 95%CI 0·41 − 0·63), chest pain (aOR 0·65, 95%CI 0·47 − 0·89), chest tightness (aOR 0·55, 95%CI 0·41 − 0·74), fatigue (aOR 0·50, 95%CI 0·41 − 0·61), muscle soreness (aOR 0·54, 95%CI 0·44 − 0·66), decreased/loss of smell (aOR 0·51, 95%CI 0·41 − 0·64), decreased/loss of taste (aOR 0·56, 95%CI 0·45 − 0·69), diarrhea (aOR 0·63, 95%CI 0·49 − 0·80) and conjunctivitis (aOR 0·48, 95%CI 0·24 − 0·95) were significantly less common among PLWH. After adjusting age, sex, BMI, comorbidities, and COVID-19 vaccination status, the risk for an increased level of fever degree (from < 37·3℃ to 37·3–38℃; from 37·3–38℃ to 38·1–39℃; from 38·1–39℃ to ≥ 39·1℃) was 0.51 times (95%CI 0·42 − 0·61) greater in PLWH than in HIV-free people, and the risk for an increased level of fever duration (from 0 days to ≤ 3 days; from ≤ 3 days to 3–5 days; from 3 to 5 days to > 5 days) was 0·52 times (95%CI 0·43 − 0·63) greater in PLWH than in HIV-free people (Fig. 1).

### Factors associated with symptoms prevalence among PLWH

The risk factors associated with the prevalence of clinical symptoms among PLWH with the Omicron variant infection were analyzed (supplementary Table 1). Males had significantly decreased risks of nasal congestion/runny nose (aOR 0·52, 95%CI 0·32 − 0·82) and headache (aOR 0·58, 95%CI 0·36 − 0·92). Older age was associated with significantly decreased risks of fever (aOR 0·97, 95%CI 0·96 − 0·99), nasal congestion/runny nose (aOR 0·97, 95%CI 0·96 − 0·99), sore/dry throat (aOR 0·98, 95%CI 0·96 − 0·99), headache (aOR 0·96, 95%CI 0·95 − 0·98) and diarrhea (aOR 0·96, 95%CI 0·94 − 0·99). Having comorbidities was associated with a significantly increased risk of fatigue (aOR 1·45, 95%CI 1·03 − 2·03). Compared with CD4 count ≥ 500 cells/µL, CD4 count between 350 ~ 499 cells/µL was associated with significantly decreased risks of fever (aOR 0·63, 95%CI 0·40 − 0·97), headache (aOR 0·61, 95%CI 0·41 − 0·91) and muscle soreness (aOR 0·57, 95%CI 0·39 − 0·84). Compared with undetectable HIV-VL, detectable HIV-VL was associated with significantly decreased risks of fever (aOR 0·56, 95%CI 0·33 − 0·94), headache (aOR 0·55, 95%CI 0·34 − 0·92), cough (aOR 0·50, 95%CI 0·31 − 0·83) and muscle soreness (aOR 0·57, 95%CI 0·39 − 0·84).

### Factors associated with fever degree and duration among PLWH

**Fig. 2:**
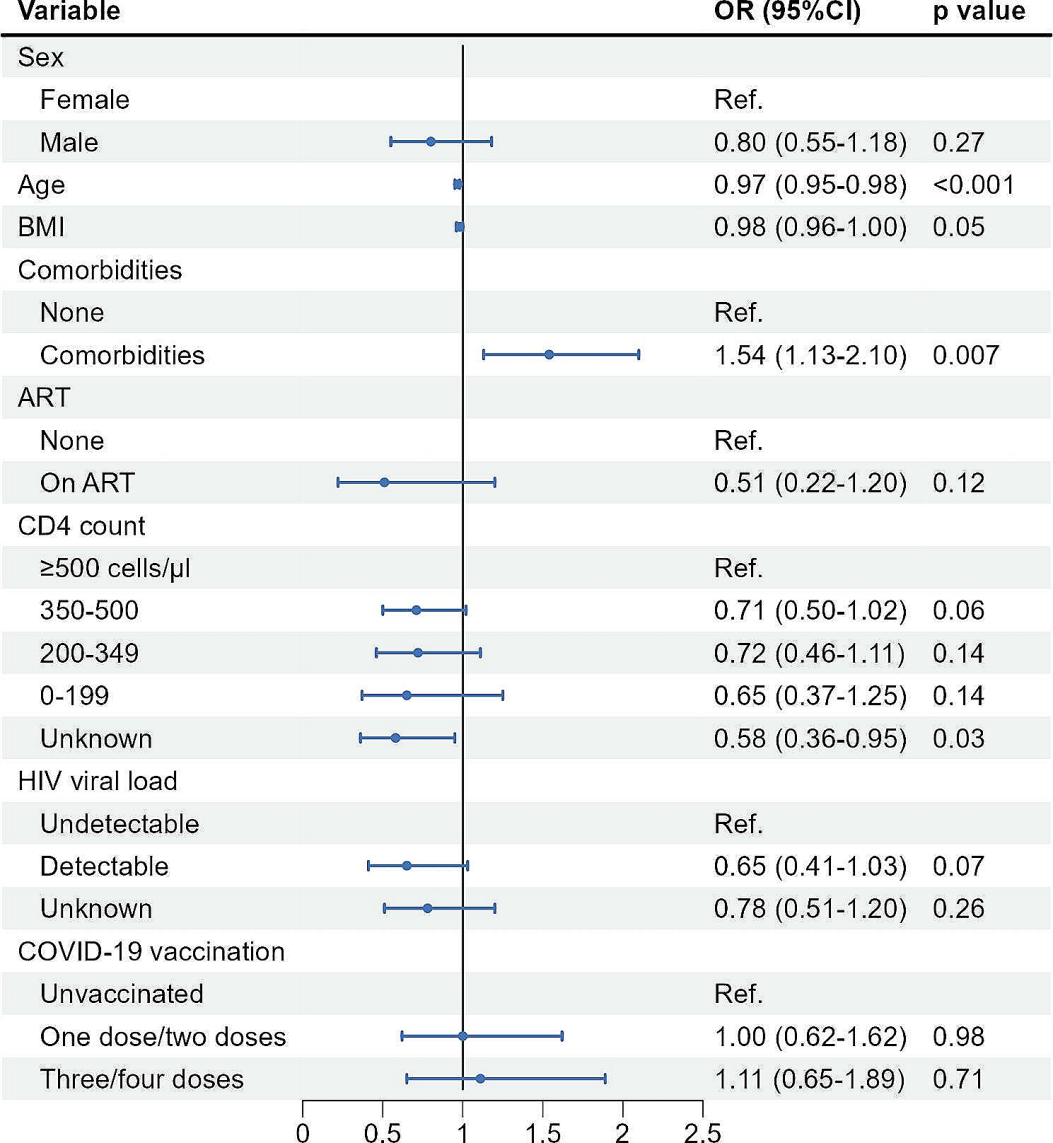
Generalized linear regression analysis of factors associated with increased level in fever degree (from < 37·3℃ to 37·3–38℃, or form 37·3–38℃ to 38·1–39℃, or from 38·1–39℃ to ≥ 39·1℃) among PLWH with SARS-CoV-2 infection

**Fig. 3:**
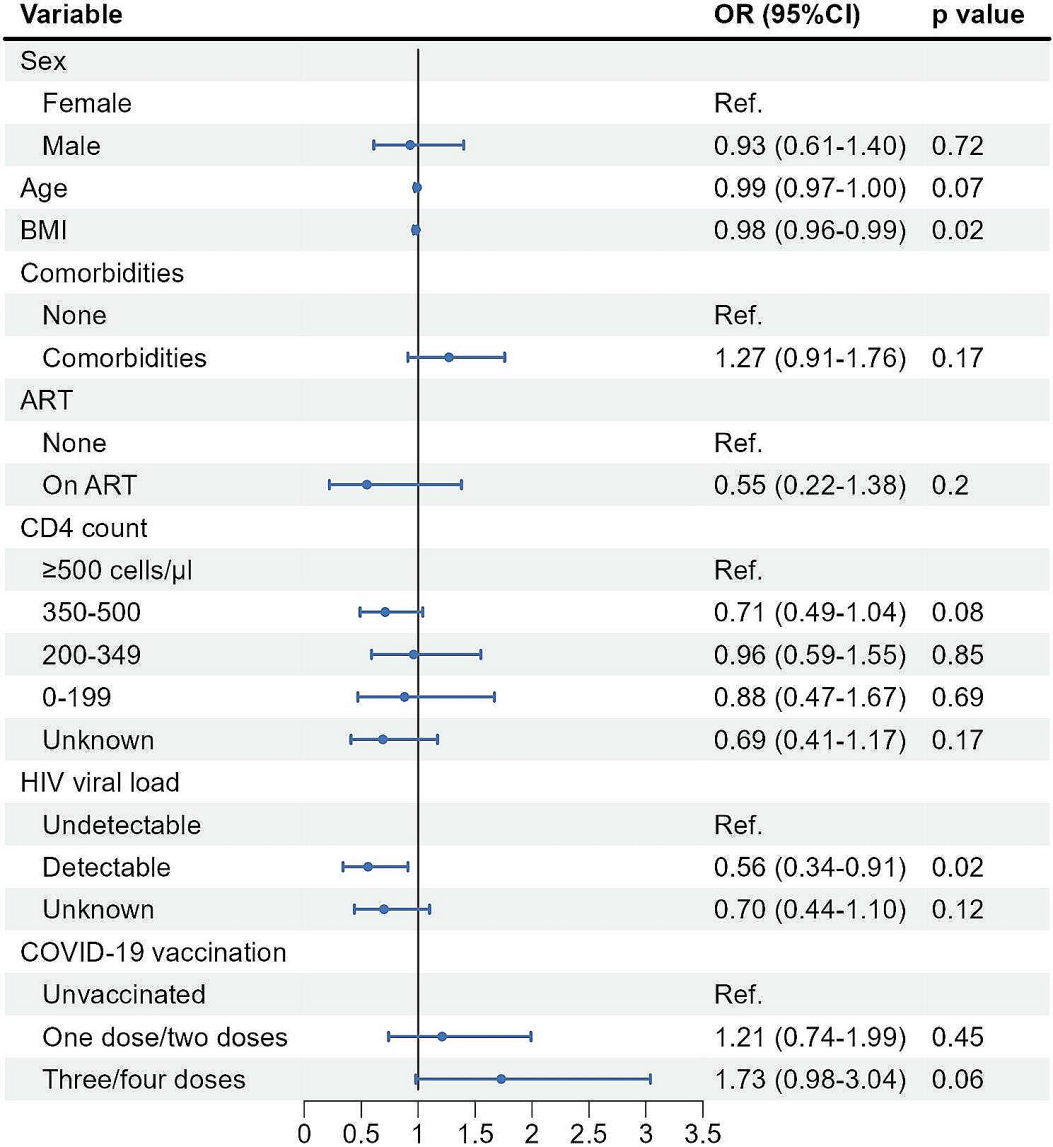
Generalized linear regression analysis of factors associated with increased level in fever duration (from 0 days to ≤ 3 days, or from ≤ 3 days to 3–5 days, or from 3–5 days to > 5 days) among PLWH with SARS-CoV-2 infection

Generalized linear regression models further analyzed the factors associated with an increased level of fever degree and an increased level of fever duration among PLWH with the Omicron variant infection. Older age was associated with a significantly decreased risk of a higher degree of fever (aOR 0·97, 95%CI 0·95 − 0·98), while having comorbidities was associated with a significantly increased risk of a higher degree of fever (aOR 1·54, 95%CI 1·13 − 2·10) (Fig. 2). Lower BMI (aOR 0·98, 95%CI 0·96 − 0·99) and detectable HIV-VL (aOR 0·56, 95%CI 0·34 − 0·91) were also associated with a significantly decreased risk of longer duration of fever (Fig. 3). No apparent association was observed between the prevalence of symptoms, including fever degree and duration, and three/four doses of inactivated COVID-19 vaccination among PLWH.

## Discussion

Realizing the clinical presentations of SARS-CoV-2 infection among PLWH during the Omicron wave is essential for planning management of COVID-19, especially during the COVID-19 surge, when healthcare services are limited. Our study contributes to the existing literature by reporting the prevalence of clinical symptoms among PLWH with the Omicron variant infection and comparing it with HIV-free people. We found that PLWH with COVID-19 had significantly milder symptoms than HIV-free people with COVID-19 during the Omicron wave in China.

Our data showed that the prevalence of having any symptom was significantly lower among PLWH than among HIV-free people with the Omicron variant infection, suggesting the prevalence of asymptomatic Omicron variant infection was higher among PLWH than among HIV-free people. One explanation may be that HIV infection-related impaired immunity could lead to longer coexistence of SARS-CoV-2 for further variation and evolution in vivo [14]. Thus, PLWH may not present with typical symptoms of SARS-CoV-2 infection due to compromised immunity [2]. These may have contributed to the high asymptomatic rate of Omicron variant infection among PLWH.

Several studies have demonstrated that PLWH had less severe COVID-19 symptoms than HIV-free people [15, 16]. Our findings further showed that PLWH with the Omicron variant infection had significantly milder symptoms than HIV-free people and a substantially lower degree and shorter duration of fever. Fever, a common phenomenon of inflammation, was related to the level of the inflammatory response to a certain extent. Fever is known to be the most frequent symptom of cytokine storms, mainly associated with the over-activation of neutrophils, monocytes, macrophages, T cells and massive release of cytokines (such as IL-6, IL-2, IFN-γ, TNF-α) [17]. One hypothesis is that low lymphocyte and CD4 count among PLWH may protect the body from SARS-CoV-2-induced cytokine storms [18]. Moreover, in SARS-CoV-2 infection, excessive activation of T cells could lead to severe immune damage in vivo [19–21]. Hence, impaired T-cell response caused by HIV may reduce the clinical severity of SARS-CoV-2 infection.

In our study, CD4 count between 350 ~ 500 cells/µL and detectable HIV-VL were associated with lower symptoms prevalence than CD4 count > 500 cells/µL and undetectable HIV-VL among PLWH with the Omicron variant infection. Our early study that summarized the characteristics of 14 patients with HIV and SARS-CoV-2 co-infection found that a relatively high CD4 count among PLWH may more easily lead to COVID-19 death [22]. This suggests that low CD4 count may be a protective factor in preventing hyperimmune response in SARS-CoV-2 infection. The viewpoint that low CD4 counts may not induce the robust inflammatory response and further reduce clinical symptoms and poor clinical outcomes of COVID-19 has been reported by some studies [23, 24]. However, there were also other studies finding that CD4 counts < 200 cells/mm3 were associated or not associated with severe clinical outcomes of COVID-19 among PLWH [25, 26]. The role and mechanism of CD4 count in the development of COVID-19 need further investigations. A retrospective cohort study in the US observed a significantly lower rate of intubations and deaths and substantially lower peak levels of immune and inflammatory markers, including CRP, IL-6, and neutrophil count among PLWH with an unsuppressed viral load than those with suppressed viral load [27]. For patients with detectable HIV-VL, impaired immune function caused by persistent HIV replication may have weakened the immune response to SARS-CoV-2, which may explain our findings.

We did not observe the apparent association between symptoms prevalence and three/four doses of inactivated COVID-19 vaccination among PLWH with the Omicron variant infection. We previously found that the third dose of inactivated COVID-19 vaccine could rapidly induce strong antibody responses among PLWH within 14 days after vaccination [10]; however, we also observed the overall immunogenicity of the third dose of inactivated COVID-19 vaccine among PLWH was still significantly lower than among healthy individuals within six months after vaccination [28]. And limited improvement in neutralizing activity of the third dose of an inactivated vaccine against the Omicron variant has been reported in other studies [29, 30]. During the Omicron variant epidemic, the third dose of inactivated COVID-19 vaccine showed weaker immunogenicity in PLWH than in healthy individuals and had a weaker ability to inhibit viral infections [31]. No significant change in the prevalence of the symptoms among PLWH with three/four doses of vaccination compared with those without vaccination may be attributed to the waning protective immunity of three/four doses of inactivated vaccines against the Omicron variant infection.

Male sex and older age have been reported to be the risk factors associated with COVID-19 mortality in patients, including those infected with HIV [32–34]. However, we found males and older age were associated with less prevalent symptoms among PLWH with the Omicron variant infection. Compared with males, the chromosomes and estrogen secretion in female sex could induce more robust neutrophils and T cells’ immune activity against viral infection, which may be related to more symptoms among females with SARS-CoV-2 infection [35, 36]. Asymptomatic SARS-CoV-2 infections have been observed to be more likely to occur in older PLWH [37]. Similarly, lower immune activity against viral infection among older people may contribute to milder symptoms in the Omicron variant infection.

Our study had some limitations. First, not all individuals with asymptomatic infection performed the SARS-CoV-2 nucleic acid testing or rapid antigen test, so the prevalence of asymptomatic infection among the population might be underestimated. Second, a selection bias may exist because only individuals with internet access participated in this online survey. But we anticipated that this would be comparable between PLWH and HIV-free people. Third, we didn’t collect the treatment data of COVID-19 therapy in our study since the anti-viral drugs against COVID-19 was unavailable in China during the study period. Finally, some symptoms reported may not be associated with SARS-CoV-2 infection. However, we have lessened this possibility by reporting the symptoms during the time or within two weeks of acute SARS-CoV-2 infection.

## Conclusions

PLWH with SARS-CoV-2 infection had significantly milder symptoms than HIV-free people during the Omicron wave. Males, older age, low CD4 counts and detectable HIV-VL may be associated with lesser symptoms among PLWH with the Omicron variant infection. Our findings would be essential for decentralized care of COVID-19.

### Electronic supplementary material

Below is the link to the electronic supplementary material.

Supplementary Material 1

## Data Availability

No datasets were generated or analysed during the current study.

## Abbreviations

COVID-19: Coronavirus disease 2019
PLWH: People living with HIV
CD4 count: CD4 + T lymphocyte count
HIV-VL: HIV viral load
SARS-CoV-2: Severe acute respiratory syndrome coronavirus 2
CDC: Center for disease control and prevention
ART: Antiretroviral therapy
aOR: Adjusted odds ratios
BMI: Body mass index
