# Supplementary material for: People living with HIV with the Omicron variant infection have milder COVID-19 symptoms: results from a cross-sectional study
Source: AIDS Res Ther. 2024 Aug 10;21:53. doi: 10.1186/s12981-024-00633-4 (PMC11316988; doi:10.1186/s12981-024-00633-4)
Supplement: Supplementary file 1 — Supplementary Material 1 [file 12981_2024_633_MOESM1_ESM.docx]

Supplementary Table 1: Factors associated with occurrence of clinical symptoms among PLWH with SARS-CoV-2 infection in Wuhan, Hubei Province, China (N=687)

| aOR (95%CI) | | | | | | | |
| --- | --- | --- | --- | --- | --- | --- | --- |
|  | Any symptom | Fever | Nasal congestion/runny nose | Sore/dry throat | Headache | Cough | Chest pain |
| Sex |  |  |  |  |  |  |  |
| Female (ref) | ·· | ·· | ·· | ·· | ·· | ·· | ·· |
| Male | 0.37(0.16-0.86) | 0.76(0.46-1.26) | 0.52(0.32-0.82) | 0.69(0.43-1.09) | 0.58(0.36-0.92) | 0.71(0.45-1.13) | 1.01(0.47-2.19) |
| Age, years | 0.97(0.96-0.99) | 0.97(0.96-0.99) | 0.97(0.96-0.99) | 0.98(0.96-0.99) | 0.96(0.95-0.98) | 0.98(0.97-1.00) | 0.97(0.95-1.00) |
| BMI, kg/m^2^ | 0.99(0.92-1.05) | 0.98(0.93-1.03) | 1.05(1.00-1.11) | 1.01(0.96-1.06) | 1.01(0.96-1.06) | 1.02(0.98-1.07) | 1.01(0.94-1.08) |
| Comorbidities |  |  |  |  |  |  |  |
| None (ref) | ·· | ·· | ·· | ·· | ·· | ·· | ·· |
| Comorbidities | 1.60(0.92-2.79) | 1.29(0.87-1.90) | 1.12(0.79-1.58) | 1.36(0.96-1.94) | 1.74(1.21-2.49) | 1.33(0.94-1.90) | 2.26(1.34-3.82) |
| ART |  |  |  |  |  |  |  |
| None (ref) | ·· | ·· | ·· | ·· | ·· | ·· | ·· |
| On ART | 0.81(0.16-4.15) | 0.43(0.13-1.44) | 1.18(0.43-3.24) | 0.99(0.37-2.64) | 0.83(0.29-2.34) | 0.62(0.22-1.78) | 0.69(0.13-3.56) |
| CD4 T count, cells/µL |  |  |  |  |  |  |  |
| ≥500 (ref) | ·· | ·· | ·· | ·· | ·· | ·· | ·· |
| 350-500 | 0.80(0.44-1.44) | 0.63(0.40-0.97) | 1.05(0.71-1.57) | 0.77(0.52-1.15) | 0.61(0.41-0.91) | 0.94(0.63-1.40) | 0.65(0.33-1.26) |
| 200-349 | 1.14(0.53-2.46) | 0.85(0.49-1.48) | 0.81(0.50-1.34) | 1.03(0.62-1.72) | 0.94(0.57-1.57) | 0.84(0.51-1.39) | 1.10(0.52-2.33) |
| 0-199 | 1.34(0.50-3.62) | 0.81(0.39-1.66) | 1.18(0.60-2.30) | 0.67(0.35-1.29) | 0.85(0.43-1.65) | 1.42(0.72-2.81) | 0.63(0.18-2.27) |
| Unknown | 1.19(0.51-2.79) | 0.67(0.36-1.23) | 0.87(0.49-1.52) | 0.74(0.42-1.30) | 0.80(0.45-1.42) | 1.19(0.67-2.11) | 0.80(0.31-2.10) |
| HIV viral load |  |  |  |  |  |  |  |
| Undetectable (ref) | ·· | ·· | ·· | ·· | ·· | ·· | ·· |
| Detectable | 0.32(0.17-0.62) | 0.56(0.33-0.94) | 0.69(0.42-1.13) | 0.63(0.38-1.03) | 0.55(0.34-0.92) | 0.50(0.31-0.83) | 0.78(0.33-1.84) |
| Unknown | 0.49(0.24-0.97) | 0.73(0.43-1.24) | 1.04(0.63-1.71) | 0.64(0.40-1.05) | 1.06(0.64-1.76) | 0.53(0.32-0.87) | 0.65(0.25-1.64) |
| (Table 3 continues on next page) | | | | | | | |
|  | Any symptom | Fever | Nasal congestion/runny nose | Sore/dry throat | Headache | Cough | Chest pain |
| (Continued from previous page) | | | | | | | |
| COVID-19 vaccination |  |  |  |  |  |  |  |
| Unvaccinated (ref) | ·· | ·· | ·· | ·· | ·· | ·· | ·· |
| One dose/two doses | 0.88(0.37-2.09) | 1.32(0.68-2.55) | 1.15(0.63-2.07) | 1.12(0.62-2.04) | 0.97(0.52-1.78) | 1.20(0.66-2.18) | 0.97(0.37-2.54) |
| Three/four doses | 1.21（0.55-2.67） | 1.06(0.60-1.89) | 1.72(1.01-2.91) | 1.32(0.77-2.24) | 0.95(0.55-1.64) | 1.42(0.84-2.41) | 0.99(0.42-2.33) |

| aOR (95%CI) | | | | | | | |
| --- | --- | --- | --- | --- | --- | --- | --- |
|  | Chest tightness | Fatigue | Muscle soreness | Decreased/lost smell | Decreased/lost taste | Diarrhea | Conjunctivitis |
| Sex |  |  |  |  |  |  |  |
| Female (ref) | ·· | ·· | ·· | ·· | ·· | ·· | ·· |
| Male | 0.70(0.37-1.33) | 0.97(0.63-1.51) | 0.86(0.55-1.34) | 1.08(0.64-1.84) | 1.05(0.65-1.70) | 0.82(0.47-1.44) | 1.88(0.22-15.80) |
| Age, years | 0.99(0.96-1.01) | 0.98(0.97-1.00) | 0.99(0.97-1.00) | 0.99(0.97-1.00) | 0.99(0.97-1.00) | 0.96(0.94-0.99) | 1.00(0.94-1.06) |
| BMI, kg/m^2^ | 1.02(0.96-1.08) | 1.00(0.96-1.05) | 0.97(0.92-1.01) | 1.02(0.97-1.07) | 0.98(0.94-1.03) | 1.01(0.95-1.06) | 1.00(0.83-1.21) |
| Comorbidities* |  |  |  |  |  |  |  |
| None (ref) | ·· | ·· | ·· | ·· | ·· | ·· | ·· |
| Comorbidities | 1.37(0.82-2.30) | 1.45(1.03-2.03) | 1.30(0.92-1.83) | 1.42(0.96-2.10) | 1.23(0.85-1.77) | 1.60(1.05-2.45) | 2.49(0.75-8.27) |
| ART |  |  |  |  |  |  |  |
| None (ref) | ·· | ·· | ·· | ·· | ·· | ·· | ·· |
| On ART | 0.33(0.10-1.04) | 0.54(0.20-1.45) | 0.48(0.17-1.35) | 0.63(0.22-1.79) | 0.50(0.18-1.39) | 1.22(0.31-4.78) | ·· |
| CD4 T count, cells/µL |  |  |  |  |  |  |  |
| ≥500 (ref) | ·· | ·· | ·· | ·· | ·· | ·· | ·· |
| 350-500 | 0.98(0.50-1.89) | 0.78(0.53-1.15) | 0.57(0.39-0.84) | 1.15(0.73-1.80) | 0.69(0.45-1.05) | 0.91(0.55-1.50) | 0.76(0.14-4.25) |
| 200-349 | 1.56(0.74-3.32) | 0.93(0.57-1.52) | 0.74(0.45-1.22) | 0.59(0.30-1.14) | 0.73(0.42-1.25) | 1.21(0.65-2.25) | 2.59(0.55-12.34) |
| (Table 3 continues on next page) | | | | | | | |
|  | Chest tightness | Fatigue | Muscle soreness | Decreased/lost smell | Decreased/lost taste | Diarrhea | Conjunctivitis |
| (Continued from previous page) | | | | | | | |
| 0-199 | 1.78(0.69-4.64) | 0.84(0.43-1.64) | 0.69(0.36-1.32） | 0.88(0.39-1.99) | 0.68(0.32-1.41） | 0.78(0.32-1.93) | 2.54(0.25-25.44) |
| Unknown | 1.71(0.75-3.90) | 1.15(0.66-2.00) | 0.80(0.46-1.38) | 1.10(0.58-2.10) | 0.67(0.36-1.25) | 0.70(0.34-1.44) | 2.03(0.27-15.24) |
| HIV viral load |  |  |  |  |  |  |  |
| Undetectable (ref) | ·· | ·· | ·· | ·· | ·· | ·· | ·· |
| Detectable | 0.95(0.44-2.04) | 0.64(0.38-1.06) | 0.47(0.28-0.77) | 0.86(0.46-1.62) | 0.93(0.54-1.62) | 0.89(0.46-1.72) | ·· |
| Unknown | 0.71(0.32-1.57) | 0.64(0.39-1.05) | 0.74(0.46-1.21) | 1.08(0.61-1.92) | 0.93(0.54-1.60) | 1.39(0.75-2.57) | 0.80(0.12-5.22) |
| COVID-19 vaccination |  |  |  |  |  |  |  |
| Unvaccinated (ref) | ·· | ·· | ·· | ·· | ·· | ·· | ·· |
| One dose/two doses | 1.16(0.46-2.94) | 0.90(0.50-1.62) | 0.71(0.39-1.30) | 1.04(0.48-2.27) | 0.99(0.49-1.99) | 0.96(0.45-2.05) | ·· |
| Three/four doses | 0.97(0.42-2.27) | 0.91(0.54-1.54) | 0.77(0.45-1.31) | 1.60(0.81-3.19) | 1.69(0.91-3.12) | 1.05(0.53-2.06) | ·· |
| PLWH=people living with HIV. aOR=adjusted odds ratio. BMI=body mass index. ART=antiretroviral therapy.  * Refer to diabetes, cardiovascular and cerebrovascular diseases (hypertension, coronary heart disease, cerebral infarction, etc.), chronic lung diseases (chronic bronchitis, asthma, etc.), kidney diseases (chronic nephritis, nephrotic syndrome, chronic kidney disease and hemodialysis, etc.), chronic liver diseases, neoplastic diseases, autoimmune diseases. | | | | | | | |
